# Monogeneans of *Colossoma macropomum* (Cuvier, 1818) (Characiformes: Serrasalmidae) farmed in the state of Acre, Amazon (Brazil)

**DOI:** 10.1590/S1984-29612022042

**Published:** 2022-08-01

**Authors:** Maralina Torres da Silva, Pedro Hercílio de Oliveira Cavalcante, Cláudia Portes Santos

**Affiliations:** 1 Instituto Federal do Acre – IFAC, Rio Branco, AC, Brasil; 2 Instituto Federal do Acre – IFAC, Xapuri, AC, Brasil; 3 Laboratório de Avaliação e Promoção da Saúde Ambiental, Instituto Oswaldo Cruz, Fiocruz, Rio de Janeiro, RJ, Brasil

**Keywords:** Fish farming, Monogenea, tambaqui, Piscicultura, Monogenea, tambaqui

## Abstract

Parasitism of *Colossoma macropomum* is of particular concern because it is the most commonly farmed native fish species in Brazil. Nevertheless, the parasitic fauna of this species in the state of Acre has been little studied. For this reason, an evaluation was made of the parasitic fauna of farmed *C. macropomum* in the municipality of Rio Branco in southwestern Amazon. Four monogenean species were found in the 122 fish examined: *Anacanthorus spathulatus*, *Linguadactyloides brinkmanni*, *Notozothecium janauachensis* and *Mymarothecium boegeri*. The most prevalent species was *A. spathulatus* (50%), followed by *N. janauachensis* (44.3%), *M. boegeri* (20.5%) and *L. brinkmanni* (9.0%). These results are the first data on the ecological indices of monogeneans in tambaqui in the state of Acre and will be useful for future comparisons of the influence of environmental factors on the parasite-host relationship.

## Resumo

O parasitismo em *Colossoma macropomum* é particularmente preocupante porque é a espécie de peixe nativa mais cultivada no Brasil, no entanto a fauna parasitária dessa espécie no estado do Acre é pouco estudada. Assim, foi avaliada a fauna parasitária de *C. macropomum* cultivado no município de Rio Branco, no sudoeste da Amazônia. Foram encontradas quatro espécies de monogeneas nos 122 peixes examinados: *Anacanthorus spathulatus, Linguadactyloides brinkmanni, Notozothecium janauachensis* e *Mymarothecium boegeri*. As espécies mais prevalentes foram *A. spathulatus* (50%), seguido de *N. janauachensis* (44,3%), *M. boegeri* (20,5%) e *L. brinkmanni* (9,0%). Esses resultados são os primeiros dados sobre os índices ecológicos dos parasitos monogenéticos em tambaqui no estado do Acre e serão úteis para futuras comparações da influência dos fatores ambientais na relação parasito-hospedeiro.

## Introduction

Current knowledge about the biodiversity of parasites in *Colossoma macropomum* (Cuvier, 1818), a Serrasalmidae popularly known as tambaqui, shows that monogeneans represent the majority of helminth species that reported parasitizing this fish species in different localities (Kritsky et al., 1979; Pamplona-Basilio et al., 2001; Fischer et al., 2003; Cohen & Kohn, 2005, 2009; Morais et al., 2009; Godoi et al., 2012; Soberon et al., 2014; Dias et al., 2015; Chagas et al., 2016; Baia et al., 2019; Fujimoto et al., 2019; Mangas et al., 2020).

Parasitism in *C. macropomum* is of particular concern because this is the most commonly farmed native fish species in Brazil (IBGE, 2019). According to Valladão et al. (2018), the species is usually farmed in intensive and super-intensive systems, which may favor the occurrence and dissemination of parasitic diseases (Jerônimo et al., 2017; Farias et al., 2021).

Studies have reported damages caused by species of the class Monogenea in farmed *C. macropomum* (Santos et al., 2013; Soberon et al., 2014; Dias et al., 2015; Mangas et al., 2020). Among these damages are displacement of gill epithelium, focal hyperplasia of epithelial cells, lamellar fusion, congestion and shortening of the secondary lamellae of gills, as well as a complete fusion of the secondary lamellae (Tavares-Dias et al., 2021). However, information is lacking when it comes to the Amazon region, especially in the state of Acre. In this study, we provide new geographic distribution and ecological indexes of the parasitic monogeneans of *C. macropomum* in culture systems that can serve as a basis of comparison for future studies.

## Materials and Methods

### Ethics statement

This study was authorized by the Brazilian Institute of Environment and Renewable Natural Resources (IBAMA, Permit No. 39106/2013), in accordance with the guidelines of the Brazilian College of Animal Experimentation (COBEA).

### Study areas and collection of parasites

The fish were obtained from Colônia Santa Maria, a fish farm specializing in the production of fingerlings, in the municipality of Rio Branco (10°03'25.3”S 67°50'54.0”W), in the state of Acre, Southwestern Amazonia, Brazil. The gills and body surface the of 122 *C. macropomum* were examined in saline medium under a stereomicroscope. The parasites were fixed in 70% ethanol or 4% formalin. The monogeneans were cleared in Berlese or Hoyer medium, and some of them were stained with Gomori trichrome and examined as permanent mounts in Canada balsam. Drawings were made with the aid of a drawing tube and redrawn using Adobe Illustrator CS6. Measurements are presented in micrometers as the range, followed by the mean in parentheses, unless otherwise stated.

Parasites were identified as proposed by Kritsky et al. (1979, 1996), Thatcher & Kritsky (1983), Belmont-Jégu et al. (2004), Cohen & Kohn (2005), Boeger et al. (2006) and Cohen et al. (2013).

The prevalence, mean intensity and mean abundance were calculated for each helminth species, according to Bush et al. (1997). The prevalence was the ratio between the number of infected animals and the total number of animals analyzed. The mean intensity was the total number of helminths of a certain species divided by the number of animals infected by this species. The dominance frequency, i.e. the percentage of the infracommunities in which a given parasite species is numerically dominant was calculated according to Rohde et al. (1995).

The dispersion index (ID) and Poulin discrepancy index (D) were employed to detect distribution patterns of the parasite infracommunity (Rózsa et al., 2000) in species with prevalence ≥10%. The dispersion index (ID) significance was tested using the d-statistic according to Ludwig & Reynolds (1988).

Specimens were deposited at the Helminthological Collection of the Oswaldo Cruz Institute (CHIOC), Brazil.

## Results

One hundred and twenty-two specimens of *C. macropomum*, measuring 4–42 (17 ± 6.2) cm in length and weighed 1–1340 (110 ± 176.1) g, were examined for the presence of monogeneans and 73 (59.8%) were found to be parasitized by at least one species. A total of 3,624 monogeneans were collected in the gills and none was found on the body surface. The parasites comprised four species of Monogenea in the new geographical location: 10°03'25.3”S 67°50'54.0”W, Rio Branco, state of Acre, Brazil. The measurements corresponding of these species are presented in Table 1. Data on prevalence, mean intensity, mean abundance and number/range of parasites are displayed in Table 2. The following species were found:

**Table 1:** Measurements of monogeneans found parasitizing *Colossoma macropomum* farmed in the state of Acre, Amazon, Brazil.

**Measurements**	***Anacanthorus*** ***spathulatus***	***Linguadactyloides brinkmanni***	***Notozothecium janauachensis***	***Mymarothecium boegeri***
**﻿Body length**	445–825 (626)	1100–1725 (146)	275–382.5 (318)	230
**﻿Body width**	90–225 (159)	360–550 (456)	70–125 (96.3)	50
**﻿Haptor length**	40–110 (70)	125–200 (158)	50–75 (60)	
**﻿Haptor width**	100–175 (126)	180–275 (218)	62–95 (80)	45
**﻿Ventral bar**			55–70 (61)	53-68 (60)
**Dorsal bar**			20–40 (27)	30-50 (44)
**Ventral anchor length**		50–63 (54)	37–50 (42)	20-28 (23)
**Ventral anchor base width**		18–25 (21)	32–45 (38)	13-23 (19)
**﻿Dorsal anchor length**		38–43 (40)	15–22 (19)	20-25 (23)
**Dorsal anchor base width**		20–25 (23)	10–17 (12)	15-23(19)
**﻿Male copulatory organ**	75–85 (78)		22–37 (28)	43-60 (49)

**Table 2:** Prevalence, mean abundance, mean intensity, total number of parasites, range of variation and dominance frequency of monogeneans of *Colossoma macropomum* farmed in the state of Acre, Amazon, Brazil.

Parasites	Prevalence (%)	Mean Abundance	Mean Intensity	Total number of parasites	Range of variation	Dominance frequency (%)
*Anacanthorus spathulatus*	50	17.1	34.1	2082	1-794	36.9
*Linguadactyloides brinkmanni*	9	0.2	2.0	22	1-5	0.8
*Notozothecium janauachensis*	44.3	8.6	19.4	1046	1-255	20.5
*Mymarothecium boegeri*	20.5	3.9	18.9	474	1-192	2.5

**Figure 1:**
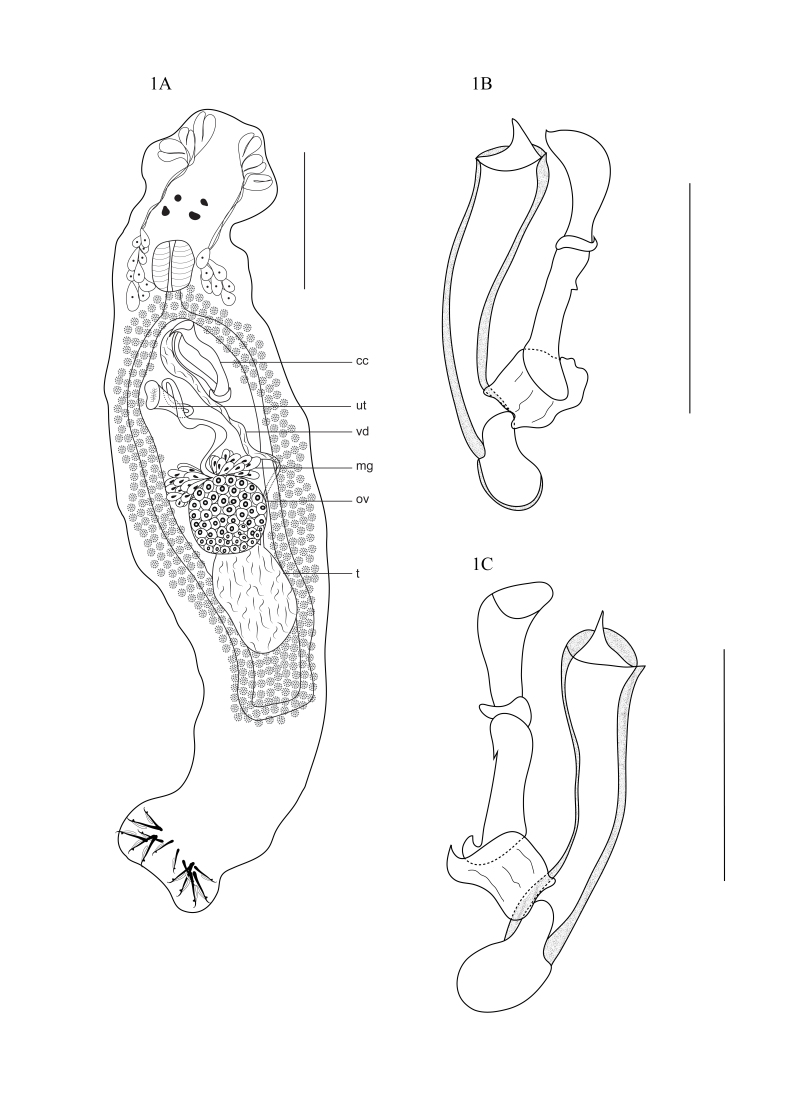
*Anacanthorus spathulatus.***1A.** Total, ventral view (cc – copulatory complex; ut – uterus; vd – ﻿vas deferens; mg – Mehlis gland; ov – ovary; t – testis). **1B.** Copulatory complex, ventral view. **1C.** Copulatory complex, dorsal view. Scale bars: A = 100 μm; B-C = 50 μm.

*Anacanthorus spathulatus* Kritsky, Thatcher & Kayton, 1979 (Figure 1ABC) (Specimens deposited: CHIOC no. 38658).

**Figure 2:**
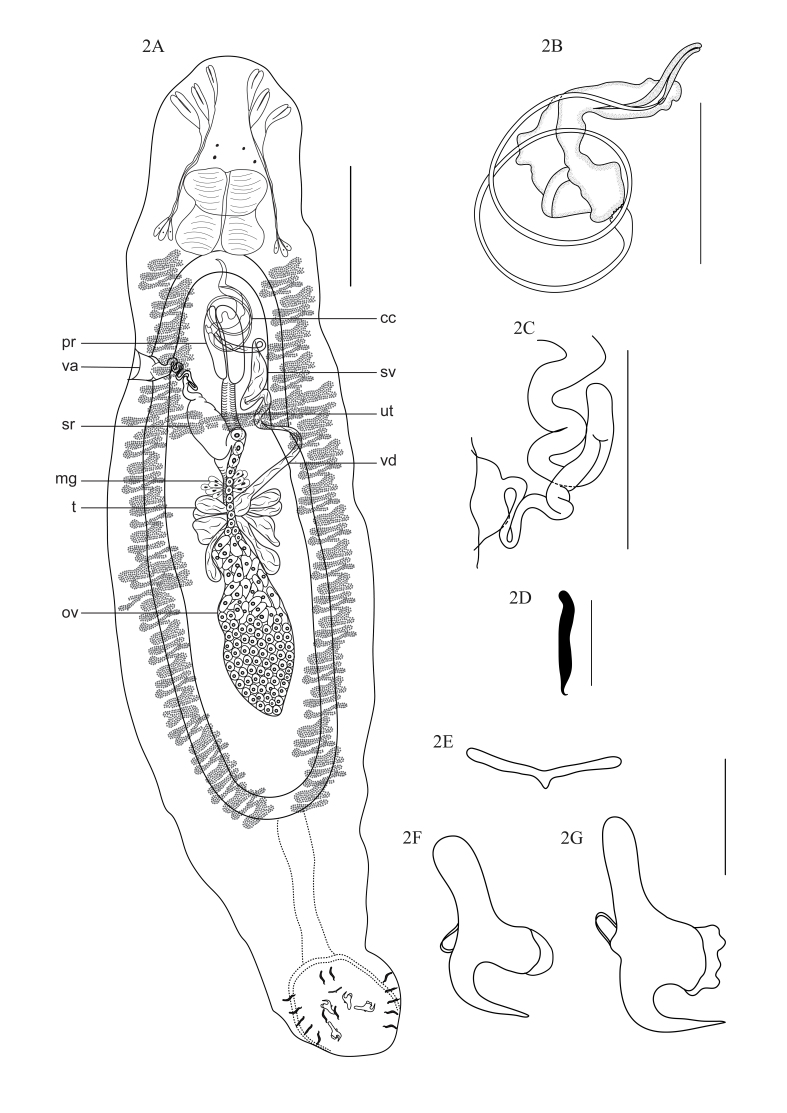
*Linguadactyloides brinkmanni.***2A.** Total view (cc – copulatory complex; pr – ﻿prostatic reservoirs; va – vagina; sv – ﻿seminal vesicle; ut – uterus; sr – seminal receptacle; vd – vas deferens; mg – Mehlis gland; t – testis; ov – ovary). **2B.** Copulatory complex. **2C.** Vagina. **2D.** Hook. **2E.** Ventral bar. **2F.** Ventral anchor. **2G.** Dorsal anchor. Scale bars: A = 200 μm; B and C = 100 μm; D–G = 25 μm.

*Linguadactyloides brinkmanni* Thatcher & Kritsky, 1983 (Figures 2A to 2G) (Specimens deposited: CHIOC no. 38663).

**Figure 3:**
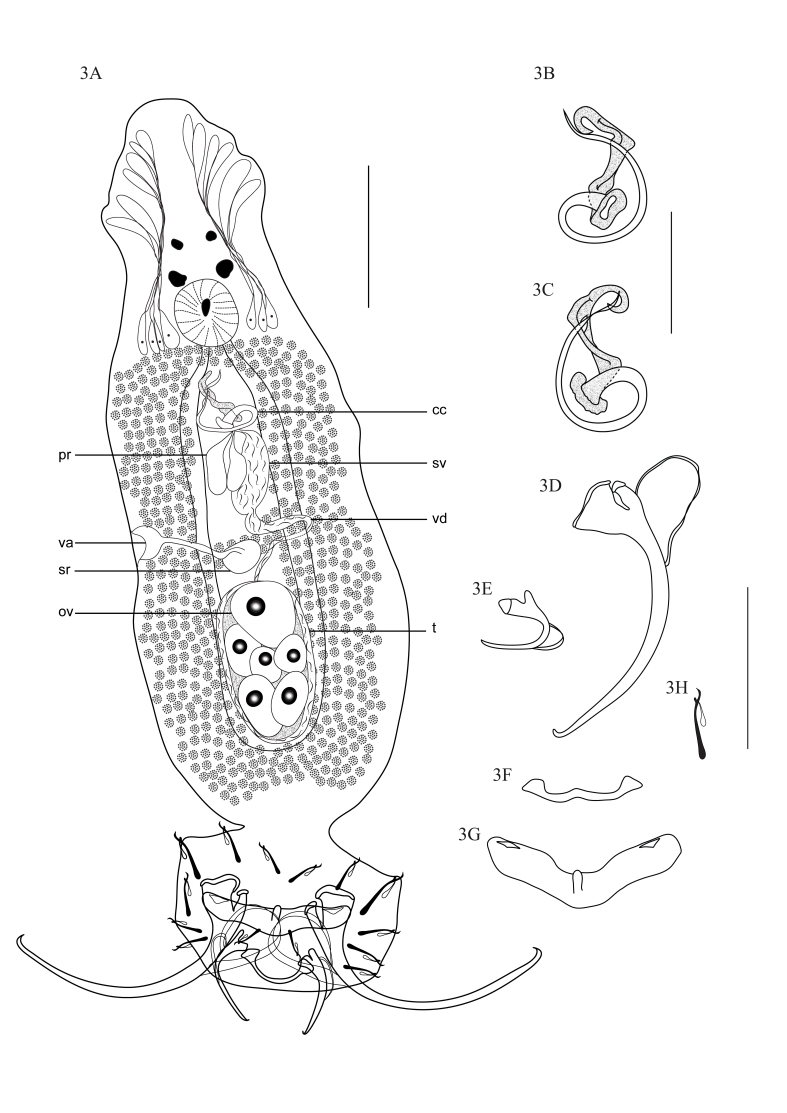
*Notozothecium janauachensis.***3A.** Total, dorsal view (cc – copulatory complex; pr – prostatic reservoirs; sv – seminal vesicle; vd – vas deferens; va – vagina; sr – seminal receptacle; ov – ovary; t – testis). **3B.** Copulatory complex, ventral view. **3C.** Copulatory complex, dorsal view. **3D.** Ventral anchor. **3E.** Dorsal anchor. **3F.** Dorsal bar. **3G.** Ventral bar. **3H.** Hook. Scale bars: A, D–H = 50 μm; B and C = 25 μm.

*Notozothecium janauachensis* Belmont-Jégu, Domingues & Martins, 2004 (Figures 3A to 3H) (Specimens deposited: CHIOC no. 38662).

**Figure 4:**
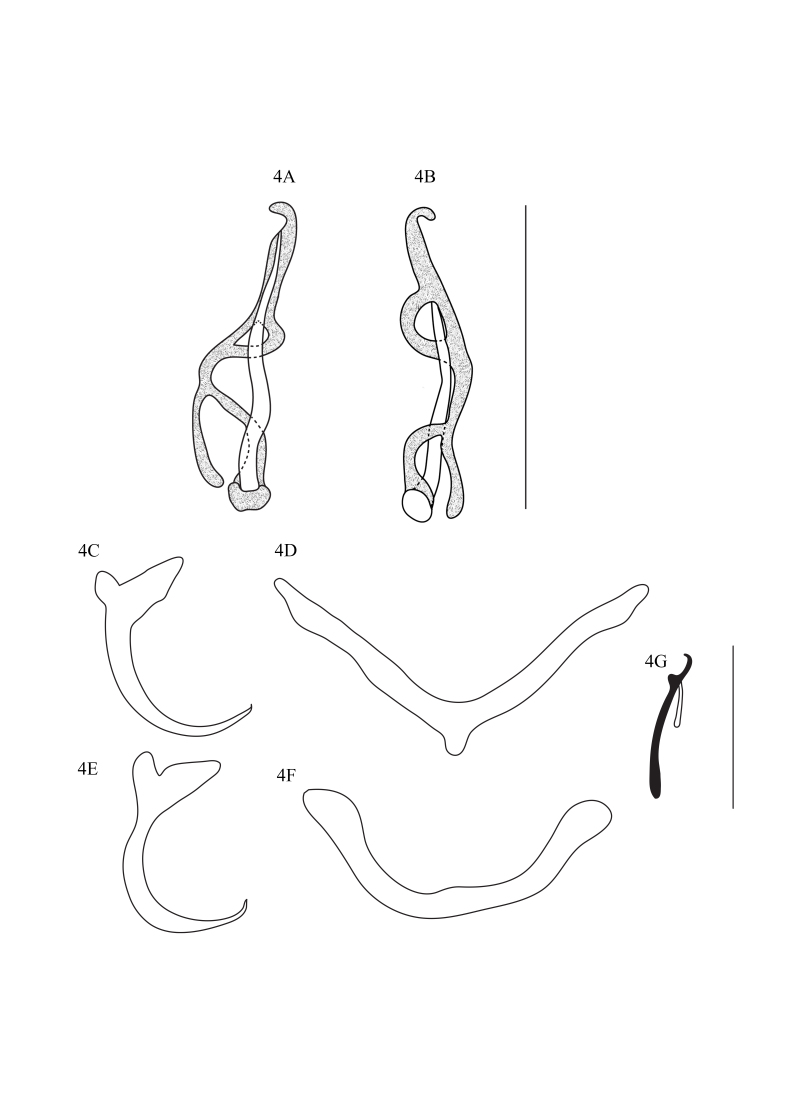
*Mymarothecium boegeri.***4A.** Copulatory complex, ventral view. **4B.** Copulatory complex, dorsal view. **4C.** Ventral anchor. **4D.** Ventral bar. **4E.** Ventral anchor. **4F.** Dorsal bar. **4G.** Hook. Scale bars: A and B = 50 μm; C–F = 25 μm.

*Mymarothecium boegeri* Cohen & Kohn, 2005 (Figures 4A to 4G) (Specimens deposited: CHIOC no. 38664).

*Anacanthorus spathulatus* was the species with higher rates of prevalence (50%), mean abundance (17.1), mean intensity (34.1) and dominance frequency (36.9%), representing 57.4% of monogeneans collected total, followed by *N. janauachensis* (Table 2).

Forty-nine fish were not parasitized (40.1%). Twenty-three fish (18.9%) were infected by only one parasitic species, with *A. spathulatus* (52.2%) being the most frequent species, followed by *N. janauachensis* (43.4%) and *L. brinkmanni* (4.3%).

Twenty-seven hosts (22.1%) harbored two parasitic species, with *A. spathulatus* and *N. janauachensis* accounting for 77.7% of double infections, followed by *A. spathulatus* and *M. boegeri* (11.1%), *A. spathulatus* and *L. brinkmanni* (7.4%), and *N. janauachensis* and *M. boegeri* (3.7%). Eighteen fish (14.8%) were parasitized by three monogenean species as follow: *A. spathulatus, N. janauachensis* and *M. boegeri* (83.3%), *A. spathulatus, N. janauachensis* and *L. brinkmanni* (11.1%) and *A. spathulatus, M. boegeri* and *L. brinkmanni* (5.5%). Five fish (4.1%) were concurrently parasitized by four monogenean species (*A. spathulatus, N. janauachensis M. boegeri* and *L. brinkmanni*).

The dispersion index (DI), statistical-*d* and discrepancy index (D) of the monogeneans of farmed *C. macropomum* showed a typical pattern of aggregated distribution (Table 3).

**Table 3:** The dispersion index (DI), statistical-*d* and discrepancy index (D) of the monogeneans of *Colossoma macropomum* farmed in the state of Acre, Amazon, Brazil*.

**Parasites**	**DI**	***d***	**D**	**Dispersion**
***Anacanthorus spathulatus***	322.72	266.89	0.857	﻿Aggregated
***Notozothecium janauachensis***	108.59	146.38	0.875	﻿Aggregated
***Mymarothecium boegeri***	94.57	134.88	0.934	﻿Aggregated

* DI and D were employed in species with prevalence ≥10%.

## Discussion

Parasitism by monogeneans is the main cause of diseases and financial losses in aquaculture (Boijink et al., 2015; Soares et al., 2016). Seven species of monogeneans are known to parasitize *C. macropomum* in fish farms in Brazil: *Anacanthorus spathulatus*, *A. penilabiatus*, *Linguadactyloides brinkmanni*, *Mymarothecium boegeri*, *M. viatorum*, *Notozothecium euzeti* and *N. janauachensis* (Cohen & Kohn, 2005, 2009; Dias et al., 2015; Chagas et al., 2016; Pamplona-Basilio et al., 2001; Silva, 2017). Four of these species (*A. spathulatus, L. brinkmanni, N. janauachensis* and *M. boegeri*) were found in this study, with morphology and measurements corresponding to those reported by Kritsky et al. (1979), Thatcher & Kritsky (1983), Belmont-Jégu et al. (2004) and Cohen & Kohn (2005), respectively.

Dias et al. (2015) and Morais et al. (2009) reported the occurrence of these same species parasitizing *C. macropomum* in fish farms in the states of Amapá and Amazonas. Chagas et al. (2016), found *A. spathulatus, N. janauachensis* and *M. boegeri* in *C. macropomum* farmed in the state of Amazonas. In the state of Rondônia, parasitism of *C. macropomum* at two fish farms included *A. spathulatus* (93 and 96%), *L. brinkmanni* (76 and 13.5%), *M. viatorum, Mymarothecium* sp. 1, *Mymarothecium* sp. 2 and *Notozothecium* spp. (79% and 94%) (Godoi et al., 2012). These data indicate a higher species diversity and higher prevalence rate of *A. spathulatus* and *Notozothecium* sp. than those found in our study. In Peruvian Amazonia, only *A. spathulatus* was reported parasitizing farmed *C. macropomum* apresented low prevalence (27.8%).

Based on the studies mentioned above, the composition of the species found in the state of Acre is similar to the species described in the neighboring states of the Brazilian Amazon (Amapá, Amazonas, Pará and Rondonia) and the Peruvian Amazon. Thus, we can infer that the component communities of the monogeneans are not isolated, but communicate through the natural evolutionary process of parasitic colonization or free movement of fry and adults between the fish farms of these states, promoting the dispersion of the monogeneans.

The presence of the same species in the different states, as well as the similar prevalence and abundance values among them, indicate that the dispersal of the monogeneans among the different localities is consistent, although further studies involving deeper analyses on the ecology and biology of this community are needed.

Furthermore, the high temperatures that remain constant throughout the year in the Amazon Region may favor the life cycle of monogenetic species, as suggested by Dias et al. (2015), Dias & Tavares-Dias (2015) and Baia et al. (2019).

Acre is among the five states with the largest deforested areas in the Brazilian Amazon, and in the coming years may suffer from long periods of drought and large forest fires (Acre, 2013; Silva et al., 2021). These environmental changes are the main causes of global warming, which among other impacts, raise the planet's temperature and affect aquatic ecosystems, especially fish, altering aspects of their physiology and increasing susceptibility to disease (Brander et al., 2018; Costa et al., 2021).

Costa et al. (2021) studied the effects of climate change on the degree of monogenetic parasitism in tambaqui. The authors concluded that increasing temperature and CO_2_ causes a rapid increase in this parasitism in seven days, which decreases in thirty days, but is still higher than in the control group. These data, indicate that special attention should be given to the culture of *C. macropomum*, especially in the state of Acre, where future forecasts indicate an increase in local average temperature (Silva et al., 2021), which may cause high rates of monogenetic parasitism, yield loss and consequent economic losses in farmed tambaqui (Costa et al., 2021).

In the present study, coexistence between monogenean species was common in 41% of hosts, which was explained by Salgado-Maldonado et al. (2019). These authors demonstrate that parasitic species can coexist in the same host population when their distributions among individual hosts are aggregated, as it occurs in aquaculture systems where high density rates are common, inevitably causing host aggregation and favoring the coexistence of parasitic species.

The aggregate distribution pattern of monogeneans of *C. macropomum* is in agreement with that reported by Gonçalves et al. (2018) in Pará state and Baia et al. (2019) in Amapá state. Poulin (2013) predicts that aggregate distributions are a common pattern in freshwater fishes and attributed the susceptibility to infection as one of the factors generating aggregation. We then conclude that as *C. macropomum* in fish farm are subject to high densities, poor management and changes in water quality, these factors make the animals more susceptible to disease occurrence and hence aggregation of parasites per host. Salgado-Maldonado et al. (2019) suggest that the greater the degree of species aggregation, the greater the intensity of infection. This positive correlation was observed in the present study, as *A. spathulatus* showed high aggregation values and high infection intensity.

Lastly, the results presented here are the first data on the ecological indices of monogenetic parasites of tambaqui in the state of Acre and will be useful for future comparisons of the influence of environmental factors on the parasite-host relationship.
